# Associations between occupational factors and self-rated health in the national Brazilian working population

**DOI:** 10.1186/s12889-019-7746-5

**Published:** 2019-10-26

**Authors:** Nágila Soares Xavier Oenning, Bárbara Niegia Garcia de Goulart, Patrícia Klarmann Ziegelmann, Jean-François Chastang, Isabelle Niedhammer

**Affiliations:** 1 Epidemiology Graduate Program, Universidade Federal do Rio Grande do Sul, Porto Alegre, Brazil; 2 INSERM, Univ Angers, Univ Rennes, EHESP, Irset (Institut de recherche en santé, environnement et travail) - UMR_S 1085, ESTER Team, Angers, France

**Keywords:** Self-rated health, Self-reported health, Workers, Working population, Occupational exposures, working conditions

## Abstract

**Background:**

The literature remains seldom on the topic of self-rated health (SRH) among the national working populations of emerging countries. The objectives of the study were to examine the associations of occupational factors with SRH in a national representative sample of the working population in Brazil.

**Methods:**

This study relied on a cross-sectional sample of 36,442 workers, 16,992 women and 19,450 men. SRH was the studied health outcome. Sixteen occupational factors related to four topics were studied: employment characteristics, working time/hours, psychosocial work factors and physical and chemical work exposures. The associations between occupational factors and SRH were studied using logistic regression models with adjustment for sociodemographic characteristics (age, ethnicity and marital status). The analyses were performed for each gender separately and using weights.

**Results:**

The prevalence of poor SRH was 26.71%, this prevalence being higher among women (29.77%) than among men (24.23%). The following risk factors for poor SRH were found among men and women: working as a self-employed worker, clerk/service worker, manual worker, part-time (≤ 20 h/week), exposure to work stress, exposure to high physical activity and exposure to sun. The risk factors for poor SRH among women only were: working as a domestic worker and exposure to noise, and among men, working in the agriculture sector.

**Conclusions:**

Our study suggested that occupational factors related to both physical and psychosocial work environment may be associated with SRH in the working population in Brazil. Improving working conditions may be beneficial for health at work in Brazil.

## Background

Self-rated health (SRH) is a measure of the general health condition as self-perceived by individuals, and can be considered as a general indicator of morbidity and a marker of future morbidity and mortality in the general population [1, 2]. SRH is recommended by WHO [3] as a low cost and easy-to-use health measurement tool in population surveys.

SRH has a multifactorial etiology and a large number of factors of different nature may play a role. As work and occupational exposures are important determinants of health, it may be crucial to identify occupational risk factors for poor SRH. There have been numerous studies exploring occupational factors in association with SRH in various working populations. Among the studied occupational factors, psychosocial work stressors occupy an important place, as almost all studies explored one or more stressors in this topic. For example, studies found the following psychosocial work factors to be associated with SRH: low control or latitude [4–10], high psychological demands [4, 6–12], job strain [13], low social support [4, 9, 10, 14], these factors being related to the job strain model, but also low reward [12, 15], effort-reward imbalance [15–17], temporary employment [5, 13, 18], job insecurity [4, 8, 11, 14, 19–21], workplace violence/bullying [8, 11, 20], organizational injustice [7, 11], or work-family imbalance [12, 20]. The study of other types of occupational exposures has been more seldom in association with SRH, as around half of the studies also explored factors related to working time/hours or to the physical work environment. Some studies reported that long working hours [5, 22, 23] or shift or night work [22] were associated with poor SRH. Some others showed that exposures related to the physical work environment, such as physical demands, ergonomic or biomechanical exposures, were associated with poor SRH [4, 6, 7, 9, 10, 13, 14, 19, 24].

Although the studies have been numerous on the associations between occupational factors and SRH, gaps remain mainly because most of the studies did not explore the occupational factors related to working time/hours and the physical work environment. Regarding the studied populations, most of the studies focused on specific working populations for example on specific occupations, sectors or areas, making the generalisation of the results difficult to national working populations. Furthermore, only half of the studies were able to examine men and women separately, leading to a lack of information about gender differences in this topic. Finally, the majority of the studies came from the more economically developed countries, in particular Europe, and information may be missing for the rest of the world, especially for Latin America.

In order to fill these gaps, our study aimed at exploring the associations between a wide range of occupational factors and SRH in a large national representative sample of the Brazilian working population.

## Methods

This study was based on the cross-sectional data of the Brazilian National Health Survey, called in Portuguese, Pesquisa Nacional de Saúde (PNS), set up in 2013 by the Brazilian Institute of Geography and Statistics (IBGE) and the health ministry [25]. As already described in one of our previous publications [26], this is a household survey among residents 18 years of age and older in Brazil. The three-stage cluster sampling included successive selections of primary sampling units, private households and residents aged 18 years or more using simple random sampling. Details on this survey were published previously [25]. Several questionnaires were used to collect data on household characteristics, and information for all household residents, and more specifically for the selected resident in each household. The total sample of selected residents included 60,202 individuals (response rate: 91.9%). For the purpose of this study, the study sample was restricted to those who were working within the week of reference, i.e. 36,442 workers, including 16,992 women and 19,450 men.

### Self-rated health (SRH)

SRH was used as a general health status measure and based on the following item: “In general, how would you rate your health?” with response categories rated on a five-point Likert scale: “very good”, “good”, “neither good nor poor”, “poor” and “very poor”. This measure is well-known as a general perceived health tool [3]. SRH was dichotomized into: good (very good, good) and poor (fair, poor and very poor). SRH was the outcome of the study.

### Occupational factors

These factors were already constructed and used in our previous publication [26]. A total of 16 variables were used to measure occupational factors that were grouped into:

- Employment characteristics: work status, occupation and economic activities that were coded using standard classifications (ISCO and ISIC respectively), and multiple job-holder (i.e. worker having more than one job).
- Working time/hours: night/shift work (one item related to night work and one filtre item related to shift work for the workers exposed to night work, making 3 categories: no exposure, night without shift work, and night with shift work) and working hours a week (collected as a continuous variable and studied in 3 categories).
- Psychosocial work factors: workplace violence (2 items on violence at the workplace from known or unknown people) and work stress (1 item on stressful work activities).
- Physical/chemical exposures (1 item each): high physical activity, chemical agents, radioactive agents, urban waste (i.e. waste, garbage and exposure related to sewage and refuse disposal, sanitation and similar activities), biological agents, marble dust, noise, and sun.

### Covariates

Four groups of adjustment variables included:

- Sociodemographic characteristics: age, ethnicity (white vs non-white, i.e. all others), and marital status.
- Health behaviours: physical activity (1 item on activity within the past 3 months), smoking (1 item on current status) and binge drinking (5 doses or more for men and 4 doses or more for women on one occasion within the past 30 days).
- Health-related variables: private health insurance plan (1 item) and disability (1 item on disability such as physical, hearing or visual disability).
- Educational level (3 categories: primary, secondary or university)

### Statistical analysis

Firstly, a description of the sample was performed for all studied variables. Differences between men and women were tested using Rao-Scott chi-square test.

Secondly, the associations of occupational factors with SRH were studied with weighted logistic regression models, using three types of models:

- Unadjusted bivariate models between each occupational factor and SRH,
- Multivariate models with all the occupational factors together (model 1),
- Multivariate models with all the occupational factors together plus sociodemographic characteristics (model 2).

The following sensitivity analyses explored the robustness of the results:

- With additional adjustment for health behaviours, private health insurance plan and disability,
- With additional adjustment for education,
- With the SRH outcome dichotomized into poor (‘poor/very poor’) versus good (‘fair/good/very good’).

We performed all the statistical analyses for each gender separately, using weights that took the sampling characteristics, non-response and calibration into account, and using SAS 9.4 software.

## Results

The prevalence of poor SRH was 26.7% (95% CI: 25.9–27.5%) among the total study sample, with a higher prevalence among women than among men: 29.8% versus 24.2% (Table 1).

**Table 1:** Description of the study population according Self-Rated Health (SRH) and other health-related variables in 2013, PNS, Brazil

	Women (*N* = 16,992)			Men (*N* = 19,450)			
	n	%	%w	n	%	%w	*p*-value
Poor Self-Rated health (SRH)	5164	30.391	29.770	4960	25.501	24.228	<.0001
Other health-related variables							
No private health insurance	11,348	66.784	63.472	13,936	71.650	68.685	0.0000
Disability	927	5.456	5.679	1281	6.586	6.204	0.2257

%: raw frequency

%w: weighted frequency

*p-*value: Rao-Scott χ2 test *p*-value for the comparison between genders

Bivariate associations are presented in Table 2. The associations were significant with poor SRH for the following factors: self-employed and domestic workers, agriculture workers, construction workers (among men only), manual and clerks/service workers, part time work, long working hours (among women only), high physical activity, exposure to chemical agents (among women only), exposure to sun, and urban waste. Several protective associations were observed with service workers (among women only), night work (among men only), exposure to radioactive agents (among women only) and biological agents (among women only). Older age, being non-white and alone (among men only) were associated with poor SRH. Smoking and physical inactivity were risk factors for poor SRH for both genders, whereas binge drinking was a protective factor. No private health insurance plan and disability were associated with poor SRH for men and women. Regarding education, primary and secondary levels were associated with poor SRH for both genders.

**Table 2:** Bivariate associations between occupational factors, covariates and Self-Rated Health (SRH) stratified by gender, 2013, PNS, Brazil

	Women (*N* = 16,992)				Men (*N* = 19,450)			
	OR	95%CI		*p-*value	OR	95%CI		*p-*value
Employment characteristics								
Work status (ref: private employee)				<.0001				<.0001
Self-employed	1.977	1.716	2.278	<.0001	1.817	1.615	2.045	<.0001
Public employee	1.143	0.956	1.368	0.1420	1.032	0.835	1.277	0.7699
Domestic worker	2.313	1.936	2.763	<.0001	2.131	1.184	3.836	0.0117
Economic activities (ref: manufacturing)				<.0001				<.0001
Agriculture	1.793	1.356	2.370	<.0001	2.743	2.229	3.376	<.0001
Construction	0.750	0.388	1.452	0.3940	1.601	1.291	1.985	<.0001
Services	0.822	0.682	0.990	0.0390	1.167	0.979	1.391	0.0839
Occupation (ref: managers/professionals)				<.0001				<.0001
Clerks/service workers	1.767	1.442	2.164	<.0001	1.584	1.265	1.984	<.0001
Manual workers	2.877	2.363	3.505	<.0001	2.334	1.903	2.864	<.0001
Technicians/associate professionals	0.986	0.721	1.348	0.9280	1.147	0.854	1.542	0.3613
Multiple job-holder	0.804	0.604	1.069	0.1330	0.833	0.648	1.072	0.1552
Working time/hours								
Working hours a week (ref: 21–44)				<.0001				<.0001
≤ 20	1.245	1.067	1.453	0.0050	1.657	1.345	2.043	<.0001
≥ 45	1.621	1.393	1.886	<.0001	1.022	0.901	1.159	0.7363
Night/shift work (ref: no)				0.8682				0.0160
Night work	0.949	0.778	1.157	0.6041	0.806	0.684	0.951	0.0106
Night work and shift work	0.958	0.549	1.672	0.8803	0.756	0.528	1.083	0.1268
Psychosocial work factors								
Work stress	1.078	0.954	1.217	0.2271	1.119	0.988	1.267	0.0772
Workplace violence	1.540	0.945	2.509	0.0833	1.263	0.833	1.917	0.2722
Physico-chemical exposures								
High physical activity	1.811	1.566	2.095	<.0001	1.532	1.364	1.721	<.0001
Chemical agents	1.270	1.083	1.488	<.0001	1.119	0.972	1.288	0.1167
Noise	1.118	0.964	1.298	0.1404	1.016	0.897	1.151	0.8048
Exposure to sun	1.936	1.642	2.283	<.0001	2.024	1.807	2.266	<.0001
Radioactive agents	0.517	0.332	0.805	0.0044	0.986	0.632	1.540	0.9522
Urban waste	1.820	1.486	2.228	<.0001	1.394	1.136	1.710	0.0015
Biological agents	0.634	0.498	0.808	0.0003	0.761	0.546	1.060	0.1064
Marble dust	1.185	0.872	1.610	0.2791	1.069	0.914	1.250	0.4054
Sociodemographic characteristics								
Age (ref: < 30)				<.0001				<.0001
30–39	1.157	0.972	1.378	0.1013	1.591	1.334	1.898	<.0001
40–49	1.877	1.562	2.257	<.0001	2.421	2.044	2.867	<.0001
≥ 50	3.000	2.511	3.586	<.0001	4.112	3.457	4.891	<.0001
Ethnicity (ref: white)	1.712	1.508	1.944	<.0001	1.453	1.294	1.632	<.0001
Marital status (ref: live alone)	1.014	0.896	1.148	0.8235	1.352	1.205	1.516	<.0001
Health-related variables								
Binge drinking	0.733	0.597	0.899	0.0035	0.86	0.754	0.98	0.0244
Smoking (ref: no)				<.0001				<.0001
Ex	1.645	1.384	1.955	<.0001	1.961	1.706	2.254	<.0001
Yes	1.831	1.548	2.164	<.0001	2.033	1.750	2.360	<.0001
No physical activity	1.596	1.387	1.836	<.0001	2.294	2.022	2.603	<.0001
No private health insurance plan	2.311	2.017	2.648	<.0001	2.292	1.981	2.652	<.0001
Disability	2.573	2.001	3.309	<.0001	2.727	2.245	3.313	<.0001
Education (ref: University)				<.0001				<.0001
Secondary	1.773	1.475	2.131	<.0001	1.451	1.173	1.795	0.0002
Primary	4.067	3.376	4.900	<.0001	3.165	2.576	3.887	<.0001

Results from weighted logistic regression analysis

Multivariate associations for women are presented in Table 3 (model 1 and 2). The associations were significant with poor SRH for the following factors: being domestic and self-employed workers, clerks/service workers and manual workers, working part time (≤20 h/week), work stress, high physical activity, sun exposure and noise. Urban waste was significant in model 1 but borderline significant in model 2.

**Table 3:** Associations between occupational factors and Self-Rated Health (SRH) adjusted for covariates in women, 2013, PNS, Brazil

	Model 1				Model 2			
Women	(N = 16,992)				(N = 16,992)			
	OR	95% CI		p-value	OR	95% CI		p-value
Employment characteristics								
Work status (ref: private employee)				<.0001				<.0001
Public employee	**1.487**	**1.228**	**1.801**	**<.0001**	1.161	0.953	1.414	0.1376
Domestic worker	**1.850**	**1.515**	**2.259**	**<.0001**	**1.534**	**1.248**	**1.887**	**<.0001**
Self-employed	**1.738**	**1.488**	**2.031**	**<.0001**	**1.369**	**1.164**	**1.611**	**0.0002**
Economic activity (ref: manufacturing)				0.1893				0.2977
Agriculture	1.111	0.800	1.543	0.5285	1.149	0.820	1.611	0.4193
Construction	0.935	0.482	1.815	0.8423	1.008	0.491	2.067	0.9832
Services	0.852	0.693	1.047	0.1283	0.884	0.713	1.097	0.2629
Occupation (ref: managers/professionals)				<.0001				<.0001
Technicians/associate professionals	1.043	0.762	1.427	0.7937	1.026	0.751	1.402	0.8713
Clerks/service workers	**1.863**	**1.515**	**2.290**	**<.0001**	**1.859**	**1.505**	**2.297**	**<.0001**
Manual workers	**2.155**	**1.724**	**2.693**	**<.0001**	**2.008**	**1.594**	**2.529**	**<.0001**
Multiple job-holder	0.833	0.611	1.135	0.2472	0.865	0.636	1.177	0.3571
Working time/hours								
Working hours (ref: 21–44)				0.0020				0.0070
≤ 20	**1.333**	**1.134**	**1.566**	**0.0005**	**1.291**	**1.098**	**1.517**	**0.0020**
≥ 45	1.136	0.965	1.337	0.1262	1.130	0.958	1.332	0.1483
Night/shift work (ref: no)				0.8589				0.8595
Night work	1.052	0.857	1.291	0.6280	1.062	0.856	1.317	0.5841
Night work and shift work	1.108	0.582	2.108	0.7553	1.037	0.544	1.977	0.9115
Psychosocial work factors								
Work stress	**1.345**	**1.183**	**1.530**	**<.0001**	**1.453**	**1.276**	**1.655**	**<.0001**
Workplace violence	1.629	0.942	2.817	0.0807	1.405	0.773	2.552	0.2643
Physico-chemical exposures								
High physical activity	**1.275**	**1.088**	**1.494**	**0.0027**	**1.284**	**1.091**	**1.510**	**0.0026**
Chemical agents	1.012	0.849	1.207	0.8909	0.996	0.829	1.197	0.9682
Noise	1.157	0.978	1.369	0.0885	**1.190**	**1.002**	**1.414**	**0.0480**
Exposure to sun	**1.338**	**1.092**	**1.641**	**0.0051**	**1.331**	**1.079**	**1.640**	**0.0075**
Radioactive agents	0.730	0.454	1.173	0.1935	0.739	0.452	1.209	0.2282
Urban waste	**1.396**	**1.095**	**1.779**	**0.0071**	1.301	0.993	1.705	0.0564
Biological agents	0.807	0.616	1.058	0.1207	0.844	0.640	1.113	0.2288
Marble dust	1.006	0.735	1.378	0.9691	1.031	0.752	1.413	0.8489

Results from weighted logistic regression analysis

Model 1: all occupational factors simultaneously

Model 2: model 1 + sociodemographic characteristics

Values in bold: significant at *p* < 0.05

Multivariate associations for men are presented in Table 4 (model 1 and 2). The associations were significant with poor SRH for the following factors: being self-employed workers, agriculture workers, clerks/service workers and manual workers, working part time (≤20 h/week), work stress, high physical activity and sun exposure. Urban waste was significant in model 1 but borderline significant in model 2.

**Table 4:** Associations between occupational factors and Self-Rated Health (SRH) adjusted for covariates in men, 2013, PNS, Brazil

	Model 1				Model 2			
Men	(*N* = 19,450)				(*N =* 19,450)			
	OR	95% CI		*p-*value	OR	95% CI		*p-*value
Employment characteristics								
Work status (ref: private employee)				<.0001				0.0776
Public employee	1.190	0.942	1.503	0.1452	0.972	0.779	1.214	0.8031
Domestic worker	1.792	0.948	3.387	0.0723	1.211	0.594	2.466	0.5985
Self-employed	**1.521**	**1.342**	**1.723**	**<.0001**	**1.172**	**1.034**	**1.328**	**0.0128**
Economic activity (ref: manufacturing)				<.0001				<.0001
Agriculture	**1.760**	**1.377**	**2.250**	**<.0001**	**1.708**	**1.337**	**2.181**	**<.0001**
Construction	1.089	0.860	1.380	0.4800	1.072	0.842	1.365	0.5735
Services	1.109	0.917	1.341	0.2852	1.157	0.950	1.408	0.1460
Occupation (ref: managers/professionals)				<.0001				<.0001
Technicians/associate professionals	1.186	0.880	1.599	0.2616	1.244	0.917	1.687	0.1609
Clerks/service workers	**1.681**	**1.327**	**2.130**	**<.0001**	**1.702**	**1.334**	**2.172**	**<.0001**
Manual workers	**1.777**	**1.417**	**2.227**	**<.0001**	**1.852**	**1.464**	**2.344**	**<.0001**
Multiple job-holder	0.909	0.700	1.182	0.4780	0.911	0.696	1.192	0.4973
Working time/hours								
Working hours (ref: 21–44)				0.0007				0.0041
≤ 20	**1.436**	**1.164**	**1.771**	**0.0007**	**1.389**	**1.112**	**1.736**	**0.0038**
≥ 45	0.931	0.817	1.061	0.2827	0.930	0.816	1.061	0.2814
Night/shift work (ref: no)				0.3238				0.3483
Night work	0.966	0.808	1.156	0.7079	0.960	0.800	1.152	0.6616
Night work and shift work	0.746	0.507	1.098	0.1378	0.760	0.521	1.109	0.1546
Psychosocial work factors								
Work stress	**1.359**	**1.184**	**1.559**	**<.0001**	**1.387**	**1.207**	**1.592**	**<.0001**
Workplace violence	1.311	0.847	2.030	0.2239	1.231	0.793	1.910	0.3540
Physico-chemical exposures								
High physical activity	**1.153**	**1.013**	**1.312**	**0.0310**	**1.223**	**1.070**	**1.397**	**0.0031**
Chemical agents	0.958	0.819	1.121	0.5924	0.976	0.830	1.148	0.7689
Noise	0.972	0.843	1.120	0.6909	0.999	0.864	1.154	0.9870
Exposure to sun	**1.398**	**1.214**	**1.610**	**<.0001**	**1.335**	**1.155**	**1.544**	**<.0001**
Radioactive agents	1.170	0.721	1.898	0.5254	1.295	0.797	2.103	0.2966
Urban waste	**1.252**	**1.007**	**1.558**	**0.0436**	1.262	0.999	1.593	0.0508
Biological agents	0.805	0.545	1.189	0.2754	0.764	0.507	1.149	0.1958
Marble dust	0.995	0.826	1.197	0.9562	1.026	0.848	1.241	0.7912

Results from weighted logistic regression analysis

Model 1: all occupational factors simultaneously

Model 2: model 1 + sociodemographic characteristics

Values in bold: significant at *p* < 0.05

Sensitivity analyses showed no change in the results after additional adjustment for health behaviours, private health insurance plan and disability, except for the exposure to noise, that was no longer significant among women. The results were also unchanged after additional adjustment for education, except noise that was not significant anymore among women. The sensitivity analysis using SRH into poor (‘poor/very poor’) versus good (‘fair/good/very good’) showed that some factors were no longer significant, which was expected given the reduced statistical power (the prevalence of poor SRH, using this definition, was 2.7% (95% CI: 2.3–3.0%) among men and 3.7% (95% CI: 3.3–4.2%) among women).

## Discussion

### Main results

Strong differences were observed between genders. Women had a higher prevalence of poor SRH than men and gender-related differences were also observed for almost all studied variables, occupational factors and covariates. Several occupational factors were associated with poor SRH among men and women: being self-employed workers, clerks/service workers, manual workers, working part time (≤20 h/week), exposure to work stress, high physical activity and sun. Gender-specific associations were also observed, for women, between working as domestic workers and exposure to noise and poor SRH, and for men, between working in the agriculture sector and poor SRH.

### Comparison with the literature

The prevalence of poor SRH was 26.7% (95% CI: 25.9–27.5%) in our study sample; 29.8% for women and 24.2% for men. Previous studies among working populations also observed gender-related differences in the prevalence of poor SRH, women having a higher prevalence than men [8, 9, 12, 14, 16, 19, 22]. The gender difference in SRH is well-known and has been found in all regions of the world [27]. Numerous factors of various nature (biological, behavioural, psychological and social) have been suspected to play a role in explaining this difference. Some authors however underlined that chronic conditions may play an important role in explaining the higher prevalence of poor SRH among women, especially musculoskeletal, mental and other pain disorders, which may be ‘less considered in favour of disorders with greater impact on mortality’ [28].

Working as a clerk/service worker or manual worker was associated with poor SRH, in line with previous studies, including studies exploring social or occupational differences in SRH, that showed that low-skilled occupational or social groups were more likely to have poor SRH [4, 13, 19, 29–31]. In China, a study showed that civil servants from government departments had significantly better SRH than workers from high-tech enterprises [32]. In contrast, in our study we did not find strong differences in SRH between private and public employees, but self-employed workers had a higher prevalence of poor SRH compared to private employees.

Part-time work was associated with poor SRH in our study, in agreement with the findings from a North American study [20]. This result might be related to a healthy worker effect, that may select healthy workers into full time jobs. However, we did not find a robust association between long working hours and SRH, in line with the results from previous studies [6, 11–14, 19, 20] but contrarily to the results observed in Korea or Japan [5, 22, 23]. Our study did not provide any significant result on the association between shift/night work and SRH. In line with our results, previous studies did not report associations between shift or night work and SRH [5, 11, 13, 14, 20]. However, one study found a significant association between shift/night work and SRH [22].

In the present study, the association between work stress and poor SRH was observed for both genders. One item (stressful work activities) was used to measure work stress. Job strain, from the job strain model, is related to the combination of high job demands and low decision latitude, and is a well-known measure of work stress. Previous studies showed significant associations of job strain or its components (high demands and low latitude) with SRH [4–13]; our findings are thus in agreement with the literature. Our study did not display a significant association between workplace violence and SRH, contrarily to some rare previous studies [8, 11, 20].

In the present study, a number of occupational physico-chemical exposures, high physical activity and exposure to sun for both genders and exposure to noise among women, were associated with poor SRH. Exposure to high physical activity was associated with poor SRH in our study. Previous studies showed similar results using various measures related to physical demands or ergonomic exposures [4, 6, 7, 9, 10, 13, 14, 19]. Only one previous study among Brazilian industrial workers was found and reported that high physical demands were associated with poor SRH [24]. To our knowledge, no previous study in the literature reported an association between exposure to sun at work and SRH. A previous study showed no association between outdoor work and SRH among US workers [20]. We found no study on the association between workplace noise and SRH. However, some previous studies included noise at work in a general measure of exposure to physical demands [19] and found significant associations between physical demands and SRH. Furthermore, environmental noise (related to traffic) was associated with poor SRH [33].

### Strengths and limitations

The study included the following strengths. It relied on a very large representative national sample of the working population in Brazil, providing reliable findings on occupational exposures and SRH in this country. Our study is also one of the first studies exploring these associations in working populations of Latin America. The response rate was high (92%) and as the survey was national and weights were available and used, a generalisation of the results may be possible to the target population, i.e. the national working population in Brazil. The statistical analyses were done for each gender separately, following the best practices [34]. Differences between genders were observed for SRH, occupational factors and covariates, as already reported in our previous publication [26], however, most of the associations between occupational factors and SRH were found to be the same for men and women. Many occupational exposures were explored, including physico-chemical exposures which are less studied in the literature than psychosocial work factors in association with SRH. The outcome (SRH) is a recognized and widely used measure of health status. In our statistical analyses, adjustment was made for sociodemographic characteristics, which are known to be associated with SRH and further adjustment for health behaviours, health-related variables and education was also made in sensitivity analyses and confirmed the results. All these strengths improved our knowledge on SRH in the Brazilian working population, an understudied population in this topic.

Our study also included a number of limitations. The study was cross-sectional, consequently no conclusion about causality can be made, and reverse causation may be possible. A healthy worker effect is also conceivable, that may lead to select healthy people at the workplace and/or at the most exposed jobs, and may lead to an underestimation of the observed associations. The occupational exposures were measured using few items and without validated scales or instruments, something that may lead to potential imprecision and a bias towards the null hypothesis. For example, for the measurement of night/shift work, no precision was given regarding the definition of exposure in the questionnaire (time schedules, for example). Another example is the measurement of work stress and workplace violence that were not based on validated questionnaires. As the selection of the studied occupational factors relied on the availability of the items in the survey questionnaire, some factors such as job insecurity, temporary employment or work-life conflict may be missing [4, 5, 8, 11–14, 18–21, 35]. Exposures and outcome relied on self-reports (common method variance), leading to a potential reporting bias and potential inflated associations.

## Conclusion

We found significant associations between various occupational factors and poor SRH, especially factors related to work status, occupation, economic activity, work stress and some physico-chemical exposures. More studies may be needed on these associations, especially among the Brazilian working population. Our study suggests that preventive measures oriented towards the reduction of occupational exposures might be beneficial for SRH among working populations. Finally, our study is an attempt to contribute to the literature by addressing these issues among the working populations of Latin America.

## Data Availability

The dataset analysed during the current study is available in the Brazilian Institute of Geography and Statistics (IBGE) repository (https://www.ibge.gov.br). The data are publically available.

## Abbreviations

IBGE: Brazilian Institute of Geography and Statistics
ISCO: International Standard Classification of Occupations
ISIC: International Standard Industrial Classification of all economic activities
PNS: Pesquisa Nacional de Saúde
SRH: Self-rated health
